# Effect of Tocilizumab in Hospitalized Patients with Severe COVID-19 Pneumonia: A Case-Control Cohort Study

**DOI:** 10.3390/ph13100317

**Published:** 2020-10-17

**Authors:** Benjamin Rossi, Lee S. Nguyen, Philippe Zimmermann, Faiza Boucenna, Louis Dubret, Louise Baucher, Helene Guillot, Marie-Anne Bouldouyre, Yves Allenbach, Joe-Elie Salem, Paul Barsoum, Arezki Oufella, Helene Gros

**Affiliations:** 1 Department of Internal Medicine, Robert Ballanger Hospital, 93600 Aulnay-Sous-Bois, France; faiza.bou@hotmail.fr (F.B.); louise.baucher@hotmail.fr (L.B.); helene.guillot@ght-gpne.fr (H.G.); marie-anne.bouldouyre@ght-gpne.fr (M.-A.B.); helene.gros@ght-gpne.fr (H.G.); 2 Research & Innovation of CMC Ambroise Paré, 92200 Neuilly-Sur-Seine, France; nguyen.lee@icloud.com; 3 INSERM, Clinical Investigations Center Paris-Est, CIC-1901, Sorbonne Université, AP.HP. Pitié-Salpétrière, 75013 Paris, France; joeelie.salem@gmail.com; 4 Department of Pharmacy, Robert Ballanger Hospital, 93600 Aulnay-Sous-Bois, France; philippe.zimmermann6@gmail.com (P.Z.); louis.dubret@ght-gpne.fr (L.D.); arezki.oufella@ght-gpne.fr (A.O.); 5 Department of Internal Medicine and Clinical Immunology, Sorbonne Université, AP.HP. Pitié-Salpétrière, 75013 Paris, France; yves.allenbach@aphp.fr; 6 Department of Cardiology, Robert Ballanger Hospital, 93600 Aulnay-Sous-Bois, France; paul.barsoum@ght-gpne.fr

**Keywords:** anti-interleukin-6, COVID-19, SARS CoV2, severe pneumonia, cytokine release syndrome

## Abstract

Tocilizumab, an anti-interleukin-6 receptor, administrated during the right timeframe may be beneficial against coronavirus-disease-2019 (COVID-19) pneumonia. All patients admitted for severe COVID-19 pneumonia (SpO_2_ ≤ 96% despite O_2_-support ≥ 6 L/min) without invasive mechanical ventilation were included in a retrospective cohort study in a primary care hospital. The treatment effect of a single-dose, 400 mg, of tocilizumab was assessed by comparing those who received tocilizumab to those who did not. Selection bias was mitigated using three statistical methods. Primary outcome measure was a composite of mortality and ventilation at day 28. A total of 246 patients were included (106 were treated with tocilizumab). Overall, 105 (42.7%) patients presented the primary outcome, with 71 (28.9%) deaths during the 28-day follow-up. Propensity-score-matched 84 pairs of comparable patients. In the matched cohort (n = 168), tocilizumab was associated with fewer primary outcomes than the control group (hazard ratio (HR) = 0.49 (95% confidence interval (95%CI) = 0.3–0.81), *p*-value = 0.005). These results were similar in the overall cohort (n = 246), with Cox multivariable analysis yielding a protective association between tocilizumab and primary outcome (adjusted HR = 0.26 (95%CI = 0.135–0.51, *p* = 0.0001), confirmed by inverse probability score weighting (IPSW) analysis (*p* < 0.0001). Analyses on mortality only, with 28 days of follow-up, yielded similar results. In this study, tocilizumab 400 mg in a single-dose was associated with improved survival without mechanical ventilation in patients with severe COVID-19.

## 1. Introduction

The pandemic of the coronavirus disease (COVID-19) started in late 2019. It quickly spread worldwide, notably in Europe [1].

It is responsible for severe pneumonia, resulting in a high rate of transfers to intensive care units (ICU) and in-patient mortality of 5% to 32% [2,3]. Severity has been related to an exaggerate immune response, the cytokine release syndrome (CRS), mediated by pro inflammatory cytokines, including interleukinIL-6, IL-12 and tumor necrosis factor α, leading to various organ dysfunction including tthe lungs, brain and heart [3]. Previously, tocilizumab, an antibody targeting IL-6 receptors proved efficient against CRS [4]. Opposing CRS may decrease further inflammatory pulmonary lesions, i.e., respiratory deterioration requiring mechanical ventilation, transfers to ICU and death [5,6].

While waiting for the results of ongoing trials studying the effects of tocilizumab on COVID-19 pneumonia, starting on 23 March 2020, we administrated off-label tocilizumab to patients with severe COVID-19 pneumonia as a compassionate use.

In the present report, we assessed the effect of tocilizumab on mortality and mechanical ventilation in a cohort of patients hospitalized for severe COVID-19 pneumonia. To mitigate selection bias, we performed a triple analysis, including propensity-score matching, Cox multivariable and inverse probability score weighting analyses, to compare patients who received tocilizumab, to those who did not.

## 2. Results

In total, 246 patients were included, with 106 patients treated by tocilizumab and compared to 140 control patients (flow-chart is presented in Supplementary Materials). Overall, 105 (42.7%) patients presented the primary outcome, with 71 (28.9%) deaths during the 28 days of follow-up. Baseline characteristics and comparisons are presented in Table 1.

### 2.1. Propensity-Score Matched Cohort

**Figure 1:**
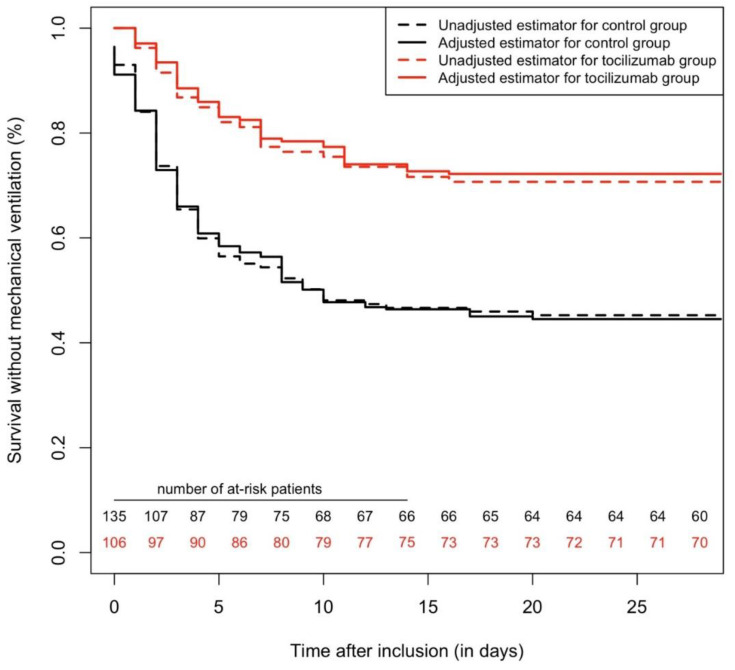
Survival curves, regarding the primary outcome with a 28-day follow-up, comparing tocilizumab and a control group. In the matched cohort (n = 168), tocilizumab was associated with fewer events (hazard ratio (HR) = 0.49 (95% confidence interval (95%CI) = 0.30–0.81), *p* = 0.005). In the overall cohort (n = 246), Cox multivariable survival analysis found tocilizumab to be independently associated with a lower incidence of the primary outcome (adjusted HR = 0.34 (95%CI = 0.22–0.52), *p* < 0.0001). Inverse probability score-weighted analysis yielded similar results (*p* < 0.0001).

Propensity-score matching yielded 84 pairs of patients (for a total of 168 patients in the matched cohort). There was no significant difference between the two matched groups regarding baseline characteristics (see Table 1). In the matched cohort (n = 168), treatment with tocilizumab was associated with fewer events (hazard ratio (HR) = 0.49 (95% confidence interval (95%CI) = 0.30–0.81), *p* = 0.005) (see Figure 1).

### 2.2. Overall Cohort (Cox Multivariable and IPSW Analyses)

In the overall cohort (n = 246), patients in the tocilizumab group were younger than those in the control group (64.3 ± 13.0 vs. 70.1 ± 16.5 year-old, *p* < 0.001), more patients under tocilizumab were labeled as having a full engagement status (73 (68.9%) vs. 71 (51.4%), *p* = 0.006), more were treated with antibiotics (106 (100%) vs. 134 (95.7%), *p* = 0.04) and by corticosteroids (43 (40.6%) vs. 38 (27.1%), *p* = 0.27) (see Table 1 for details on overall cohort). In the tocilizumab group, delay between study inclusion and tocilizumab administration was 1.0 ± 1.0 day.

Cox multivariable survival analysis found tocilizumab to be independently associated with a lower incidence of the primary outcome (adjusted HR (adj.HR) = 0.34 (95%CI = 0.22–0.52), *p* < 0.0001). Other variables associated with the primary outcome were: SpO_2_/FiO_2_ ratio on the day of inclusion (per 1 unit increase, adj.HR = 0.987 (95%CI = 0.983–0.991), *p* < 0.0001) and chronic kidney disease (adj.HR = 1.63 (95%CI = 1.03–2.52), *p* = 0.035). IPSW confirmed the protective association between tocilizumab and the primary outcome (*p* < 0.0001) (see Figure 1).

### 2.3. All-Cause Mortality (28 Days Maximum Follow-Up)

**Figure 2:**
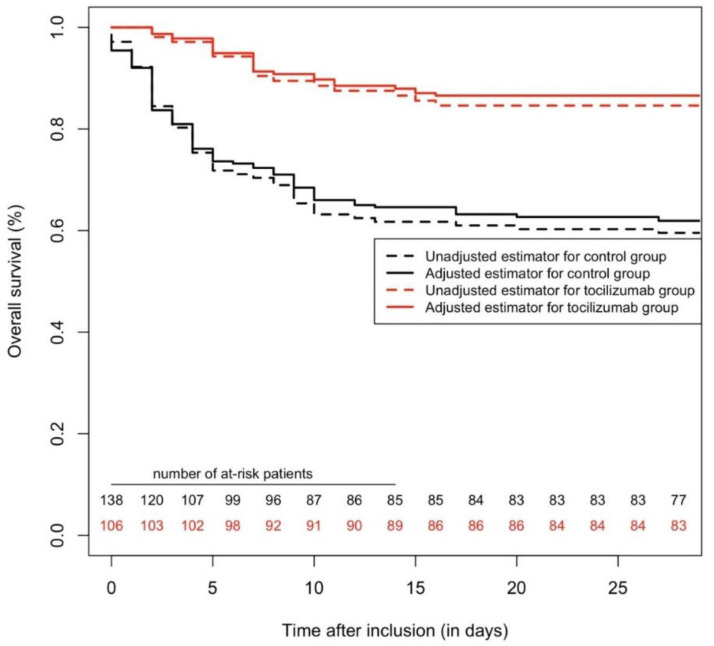
Survival curves, regarding mortality with a 28-day follow-up, comparing tocilizumab and a control group. In the matched cohort (n = 168), tocilizumab was associated with fewer deaths (hazard ratio = 0.42 (95%CI = 0.22–0.82), *p* = 0.008). In the overall cohort (n = 246), Cox multivariable analysis yielded an independent protective association between tocilizumab and mortality (adjusted HR = 0.29 (95%CI = 0.17–0.53), *p* < 0.0001). Inverse probability score-weighted analysis yielded similar results (*p* < 0.0001).

When considering all-cause mortality with a maximum follow-up of 28 days, in the matched cohort (n = 168), tocilizumab was associated with fewer deaths (HR = 0.42 (95%CI = 0.22–0.82), *p* = 0.008). In the overall cohort (n = 246), Cox multivariable analysis yielded an independent protective association between tocilizumab and mortality (adj.HR = 0.29 (95%CI = 0.17–0.53), *p* < 0.0001), the other independent variables associated with mortality were full engagement status (adj.HR = 0.11 (95%CI = 0.05–0.23), *p* < 0.0001), S/F ratio at inclusion (per 1-unit increase, adj.HR = 0.99 (95%CI = 0.985–0.995), *p* < 0.0001), chronic kidney disease (adj.HR = 2.0 (95%CI = 1.22–3.27), *p* = 0.006) and systolic blood pressure at inclusion (per 1-mmHg increase, adj.HR = 1.016 (95%CI = 1.005–1.028), *p* = 0.006). IPSW analysis was concordant (*p* < 0.0001) (see Figure 2).

### 2.4. Sensitivity Analyses

As the first sensitivity analysis, we focused on the subgroup of patients with a full engagement status. To do so, we excluded all patients labelled as “not to be admitted in ICU” or “not to be mechanically ventilated”. This analysis retained 145 patients, including 73 (50.3%) treated with tocilizumab. In these 145 patients, 43 patients presented the primary outcome. Cox multivariable analyses yielded a protective association between tocilizumab and the primary outcome (adj.HR = 0.43 (95%CI = 0.22–0.81) *p* = 0.01); the other variables independently associated with the primary outcome were the SpO_2_/FiO_2_ ratio at inclusion (per 1-unit increase, adj.HR = 0.985 (95%CI = 0.979–0.992) *p* < 0.0001) and the temperature at inclusion (per 1 °C-increase, adj.HR = 1.39 (95%CI = 1.088–1.771), *p* = 0.008). Similar to the primary outcome, focusing on mortality only, in these 145 patients, 9 died during the 28-day follow-up. Cox multivariable analyses also yielded a protective association between tocilizumab and the mortality (adj.HR = 0.11 (95%CI = 0.01–0.89) *p* = 0.039).

**Figure 3:**
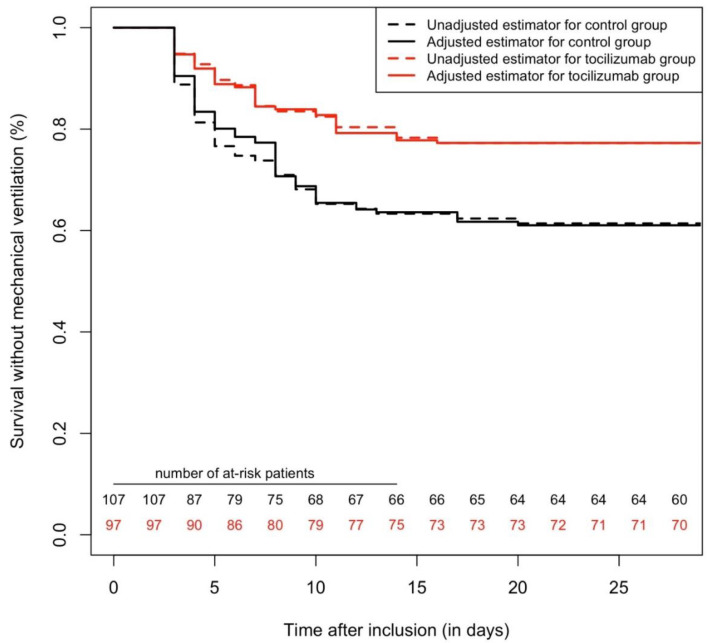
Survival curves, regarding the primary outcome with a 28-day follow-up, comparing tocilizumab and a control group, after excluding patients who presented outcomes in the first 48 h after inclusion. Cox multivariable analysis yielded a protective association between tocilizumab and the primary outcome (adj.HR = 0.40 (95%CI = 0.23–0.70), *p* = 0.001).

**Figure 4:**
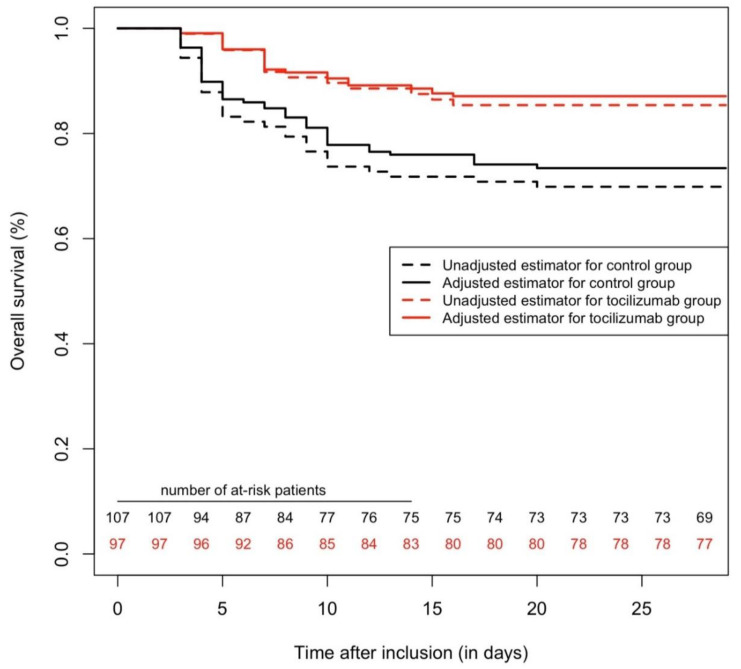
Survival curves, regarding mortality with a 28-day follow-up, comparing tocilizumab and the control group, after excluding patients who presented outcomes in the first 48 h after inclusion. Treatment by tocilizumab was found to be protectively associated with mortality (adj.HR = 0.36 (95%CI = 0.18–0.70) *p* = 0.003).

In a second sensitivity analysis, we excluded patients who presented an outcome during the first 48 h, to mitigate other selection bias (including immortal time bias). This dataset comprised 204 patients, with 97 treated by tocilizumab; 63 patients presented the primary outcome, and 46 died during the 28-day follow-up. Using a triple statistical method as in the main analysis, tocilizumab was found to be significantly associated with fewer primary outcomes (Cox multivariable analysis yielded adj.HR = 0.40 (95%CI = 0.23–0.70), *p* = 0.001) (see Figure 3). Similarly, tocilizumab was found to be protectively associated with mortality (adj.HR = 0.36 (95%CI = 0.18–0.70) *p* = 0.003) (see Figure 4) (details of the multivariable models are presented in Supplementary Materials).

## 3. Discussion

As the main finding of this single-center retrospective study, which focused on 246 patients hospitalized for severe COVID-19 pneumonia, we observed a protective association between treatment by tocilizumab and clinical outcomes, which included deaths and invasive mechanical ventilation, at 28-days of follow-up.

The study cohort was similar to that of previously described COVID-19 patients with a median age of 68 years; 27.6% presented cardiovascular history and 30.1% were obese [7]. Median delay between first symptoms and treatment was 8 days, corresponding to the delay of CRS onset described in SARS-Cov-2 [8].

Attenuating CRS may partly explain the significant decrease in the primary outcome [5,6]. Several therapeutic interventions (corticosteroids, interleukin-1 blockade) have been used to mitigate inflammatory organ injury in viral pneumonia [9]. The recent preliminary results from the RECOVERY trial provides evidence that treatment with dexamethasone reduces mortality in patients with COVID-19 under respiratory support. This study demonstrates the clinical relevance of the strategy based on inflammatory regulation in severe COVID-19 pneumonia [10].

Indeed, CRS was related to interleukin accumulation, and a recent randomized trial studying dexamethasone yielded significant benefits [11], comforting the role of immunomodulation therapeutic strategies [9]. In our study, clinical improvement observed in patients treated by tocilizumab was akin to that described in two observational studies [12,13], the most recent describing 544 patients with severe COVID-19 pneumonia criteria, the beneficial effect of tocilizumab (n = 179 in this group), with an adj.HR of 0.61 (95%CI = 0.40–0.92), regarding the same endpoint as in our study [13]. In these two studies, similar to currently enrolling randomized controlled trials, tocilizumab dosage was higher: 8 mg/kg (up to 800 mg) in one to three injections. In comparison, in our study, dosage of tocilizumab was 400 mg, injected once. Further confirming our results with the same dosage would improve the availability of this costly biotherapy for which access may become an issue.

The preliminary results of the COVACTA trial, testing the effect of tocilizumab in randomized control trial in a heterogeneous cohort of patients, did not show significance of the primary composite endpoint nor secondary mortality endpoint. However, the proportion of patients under mechanical ventilation or in ICU who were excluded in our observational study, is unknown as of now. Moreover, previous observational study protocols mentioned tocilizumab dosage as high as 800 mg twice [12,13], similar to that of ongoing trials with two injections of up to 800 mg each, within a 3-day period [14,15]. In comparison, in our study, dosage of tocilizumab was 400 mg, injected once, following previous reports of improved outcomes of chimeric antigen receptor-T-cell-induced CRS with an 8 mg/kg dosage [4]. Further confirming our results with the same dosage would improve the availability of this costly biotherapy for which access may become an issue [16].

Indeed, in our hospital, reasons for injecting only one dose of 400 mg tocilizumab was mainly driven by cost issues and difficulties in procuring this treatment. It was only made available through the extensive work of our Pharmacology department, even more so that the time of administration corresponded to the first peak of the pandemic in Europe and France in particular. Previous pharmacokinetic and pharmacodynamic analyses of tocilizumab treatment yielded an equivalence between weekly subcutaneous injections of a lower dosage, as compared to intravenous injections once per month, to treat rheumatoid arthritis [17]. Contrary to this chronic disease, COVID-19 may be assimilated to an acute infection and thus, may not require an anti-IL6 effect as prolonged as in rheumatoid arthritis, which may explain how clinical efficacy was obtained so quickly in the present study.

We acknowledge several limitations. First, the single-center nature of this study requires external validation; however, it guarantees homogeneity in the care of all patients, in our non-ICU departments dedicated to treat COVID-19 patients, i.e., observed differences are more likely to be due to tocilizumab. Second, although we aimed to mitigate selection bias using three statistical methods, including propensity-score matching, Cox multivariable and IPSW analyses, residual confounders are plausible [18,19]. Due to comorbidities and the lack of beds in the context of the COVID-19 pandemic, a significant proportion of patients were not labeled as having a full-engagement status; hence options were limited regarding the possibilities of being transferred to a critical care medical department, as well as invasive mechanical ventilation. However, these criteria did not alter indications for tocilizumab, as nearly 33% were limited at admission. Furthermore, we acknowledge that limitation of care is more granular than a binary categorical variable such as “not-to-be-resuscitated”, and involves more grades. In this retrospective study, the status of patients was hard to represent accurately, the number one reason for this being that their status evolved through time. Indeed, patients who were at first not to be ventilated, after the first few days, may have changed to be in full engagement, due to signs of improvement. Similarly, patients who were not labeled as limited when they were admitted to medical wards may have been limited during night shifts by the intensivist on duty based on comorbidities or evolution since admission. Accurately representing these variations in a simple model was not feasible, hence, we opted for the most pragmatic approach we had at our disposal: assessing when patients were flagged as full-engagement, as opposed to others. All analyses were adjusted for this criterion in multivariable models. Furthermore, we performed additional sensitivity analyses to further mitigate selection bias; analyses which yielded similar results with significant association between tocilizumab and better survival without mechanical ventilation, even focusing on patients with full therapeutic engagement (n = 155).

Third, because arterial partial pressure of O_2_ was not available in all patients, we used a SpO_2_/FiO_2_ ratio to assess respiratory dysfunction, a validated marker in acute lung injury [20]. Fourth, use of non-invasive ventilation and high-flow oxygen support changed during the study period, following evolving guidelines which advocated against doing so during the first weeks of the pandemic to decrease virus aerosol propagation, and were then made more flexible. The relatively low proportion of patients who benefited from these treatments and the fact that there was no difference between the two groups regarding this criterion decreases the chances of it being a bias, although, residual confounding biases may remain. Fourth, there was a mild difference in age between tocilizumab and control groups. However, age was a variable that was accounted for in all multivariable analyses and also in computing the propensity-score. Thus, we did not observe any interaction between age on the efficacy of treatment by tocilizumab. Finally, we did not systematically assay IL-6, which may have proven valuable to identify patients for whom the effect was greater [6].

These results point towards a clinical benefit of tocilizumab; however, they may not replace a fully-fledged randomized controlled trial, focusing on dosage adjustment and on patients managed early, so that CRS may be adequately attenuated in patients with COVID-19 evolving towards clinical deterioration.

## 4. Materials and Methods

### 4.1. Study Population

In this observational single-center study, in a primary care center regional hospital, all patients were screened for COVID-19 starting on 14 March 2020. Diagnosis required positive testing with reverse transcription polymerase chain reaction (RT-PCR) or chest CT-scan with typical lesions [21]. Inclusion criteria was severe COVID-19 pneumonia. Severity criteria required a pulse oxygen saturation (SpO_2_) ≤ 96% despite oxygen support ≥ 6 L/min with oxygen mask, for more than 6 h. We excluded patients with invasive mechanical ventilation (i.e., intubated) and those in the critical care medicine department.

### 4.2. Study Design

Starting on 23 March 2020, tocilizumab was made available for off-label compassionate use in severe COVID-19 pneumonia in our center. Patients were compared between two groups: those who received tocilizumab (a single intravenous injection, 400 mg) and those who did not (henceforth called the control group, albeit this was not a randomized controlled trial). This retrospective study was approved by a research ethics committee and was registered on clinicaltrials.gov (NCT04366206).

### 4.3. Treatment Intervention

Tocilizumab was made available for compassionate use by hospital pharmacists on 23 March 2020. The choice and indication of treatment depended on an attending physician, and information was given to all patients prior to being treated. No criteria were retained to exclude patients from treatment and patients who were not labeled as being in a full-engagement status by intensivists or attending physicians were still eligible for tocilizumab treatment.

### 4.4. Study Variables

The primary outcome was a composite of all-cause mortality and invasive mechanical ventilation (i.e., requiring tracheal intubation). The follow-up period was 28 days after inclusion. All data were prospectively collected in electronical medical records, which were then extracted for the purpose of this study. A list of the available data is presented in Supplementary Materials. Follow-up was completed for all patients.

### 4.5. Statistical Analyses

The baseline characteristics of patients treated by tocilizumab were compared to that of the control group. In the primary analysis, 1:1 nearest-neighbor propensity-score matching was performed using the following variables: age; sex; smoking status; history of coronary artery disease; stroke; heart failure or peripheral artery disease; hypertension; chronic kidney disease with eGFR less than 60 mL/min/1.73 m^2^; cancer; long-term corticosteroid treatment; use of antibiotics, of antivirals, of corticosteroids or of baricitinib after admission; SpO_2_/FiO_2_ ratio at admission; time between admission and inclusion; andSpO_2_/FiO_2_ ratio and CRP at inclusion [18]. A second analysis using a multivariable Cox proportional hazard analysis was performed in the entire cohort, with the following independent covariables: tocilizumab injection, engagement status, age, systolic blood pressure at inclusion, SpO_2_/FiO_2_ ratio at admission and SpO_2_/FiO_2_ ratio at inclusion. A third analysis using an inverse probability score weighting (IPSW) approach was also performed using the entire cohort [19]. Kaplan-Meier curves were used to compare the two groups in the matched cohort. Cox proportional hazards modeling was used to assess the association between tocilizumab and the primary outcome in the overall cohort. Conclusions were drawn only if the three analyses yielded concordant results. Continuous variables were presented as mean ± standard deviation and categorical variables as a number (proportion). All analyses were performed using SPSS 25.0 (IBM, Armonk, NY, USA) and R software version 3.6.

## 5. Conclusions

In this observational single-center study, in hospitalized patients presenting with severe COVID-19 pneumonia, a single dose of tocilizumab 400 mg was associated with lower mortality and a lesser need for mechanical ventilation.

## Figures and Tables

**Table 1:** Baseline characteristics, at admission and at inclusion.

	Control Group (n = 140)	Tocilizumab Group (n = 106)	*p*-Value	Matched Control (n = 84)	Matched Tocilizumab Group (n = 84)	*p*-Value
**Clinical Features, No (%)**						
Age, mean ± Standard deviation (SD), years	70.1 ± 16.5	64.3 ± 13.0	0.003 *	64.4 ± 16.9	64.8 ± 12.8	0.88
Female	59 (42.1%)	36 (34.0%)	0.19 ^†^	35 (41.7%)	29 (34.5%)	0.24 ^†^
Full engagement	72 (51.4%)	73 (68.9%)	**0.006 ^†^**	56 (66.7%)	58 (69%)	0.74 ^†^
Diabetes	53 (37.9%)	48 (45.3%)	0.24 ^†^	31 (36.9%)	35 (41.7%)	0.53 ^†^
Insulin treatment	23 (16.4%)	17 (16.0%)	0.93 ^†^	12 (14.3%)	9 (10.7%)	0.48 ^†^
Obesity (BMI > 30 kg/m^2^)	40 (28.6%)	34 (32.1%)	0.55 ^†^	26 (31.0%)	27 (32.1%)	0.87 ^†^
Hypertension	79 (56.4%)	64 (60.4%)	0.53 ^†^	47 (56.0%)	47 (56.0%)	1 ^†^
ACEI treatment	21 (15.0%)	16 (15.1%)	0.98 ^†^	11 (13.1%)	12 (14.3%)	0.82 ^†^
ARB treatment	22 (15.7%)	28 (26.4%)	**0.039 ^†^**	14 (16.7%)	20 (23.8%)	0.25 ^†^
History of cardiovascular disease, stroke, peripheral artery disease, heart failure	43 (30.7%)	25 (23.6%)	0.22 ^†^	21 (25.0%)	21 (25.0%)	0.1 ^†^
Smoker (active or past)	29 (20.7%)	33 (31.1%)	0.06 ^†^	21 (25.0%)	23 (27.4%)	0.73 ^†^
History of COPD, asthma, emphysema, fibrosis	22 (15.7%)	17 (16.0%)	0.369 ^†^	12 (14.3%)	15 (17.9%)	0.53 ^†^
eGFR < 60 mL/min/1.73 m^2^	24 (17.1%)	17 (16.0%)	0.82 ^†^	13 (15.5%)	12 (14.3%)	0.83 ^†^
Solid organ transplantation	3 (2.1%)	0 (0.0%)	0.26 ^‡^	2 (2.4%)	0 (0.0%)	0.5 ^‡^
HIV	1 (0.7%)	1 (0.9%)	1 ^‡^	1 (1.2%)	0 (0.0%)	1 ^‡^
Immunosuppressant drugs	12 (8.6%)	5 (4.7%)	0.24 ^‡^	4 (4.8%)	4 (4.8%)	1 ^‡^
Long-term oral corticosteroids	13 (9.3%)	5 (4.7%)	0.17 ^‡^	6 (7.1%)	4 (4.8%)	0.75 ^‡^
Malignancy (active)	17 (12.1%)	6 (5.7%)	0.08 ^†^	7 (8.3%)	5 (6.0%)	0.55 ^‡^
**Treatments after Admission**						
No.(%) under antibiotics	134 (95.7%)	106 (100.0%)	0.04 ^‡^	84 (100.0%)	84 (100.0%)	NA
Betalactamin	129 (92.1%)	105 (99.1%)	0.13 ^‡^	81 (96.4%)	83 (98.8%)	0.62 ^‡^
Macrolide	98 (70.0%)	93 (87.7%)	**0.001 ^†^**	64 (76.2%)	75 (89.3%)	0.025 ^†^
Others	21 (15.0%)	7 (6.6%)	**0.04 ^†^**	15 (17.9%)	6 (7.1%)	0.036 ^†^
No.(%) under antiviral therapy	106 (75.7%)	88 (83.0%)	0.16 ^†^	68 (81.0%)	68 (81.0%)	1 ^‡^
Hydroxychloroquine	100 (71.4%)	88 (83.0%)	0.034 ^†^	62 (73.8%)	68 (81.0%)	0.27 ^†^
Lopinavir/ritonavir	8 (5.7%)	1 (0.9%)	0.08 ^‡^	8 (9.5%)	1 (1.2%)	**0.03 ^‡^**
Immunosuppressants and/or corticosteroids	47 (33.6%)	43 (40.6%)	0.26 ^†^	24 (28.6%)	26 (31.0%)	0.74 ^†^
Baricitinib	18 (12.9%)	1 (0.9%)	**0.001 ^‡^**	0 (0.0%)	1 (1.2%)	1 ^‡^
**Characteristics at Hospital Admission**						
Delay between first symptoms and admission, means ± SD, days	5.4 ± 8.8	4.8 ± 15.3	0.74 *	5.2 ± 9.8	6.5 ± 4.9	0.29 *
SpO_2_/FiO_2_ ratio, mean ± SD	327.4 ± 108.7	338.4 ± 88.6	0.39 *	330.3 ± 108.6	341.8 ± 87.1	0.45 *
SpO_2_, mean ± SD, %	93.9 ± 3.6	94.0 ± 3.9	0.81 *	94.0 ± 3.6	94.2 ± 3.3	0.62 ^§^
Oxygen flow, mean ± SD, L/min	4.0 ± 4.8	3.1 ± 3.6	0.44 ^§^	3.9 ± 4.6	3.0 ± 3.3	0.54 ^§^
PaO_2_, mean ± SD, mmHg	70.4 ± 24.7	68.6 ± 17.5	0.54 *	71.6 ± 28.4	68.6 ± 17.8	0.46 *
Temperature, mean ± SD, °C	37.2 ± 1.1	37.5 ± 1.2	0.06 *	37.3 ± 1.1	37.5 ± 1.2	0.22 *
C-reactive protein, mean ± SD, mg/L	132.1 ± 99.2	135.2 ± 88.2	0.79 *	138.2 ± 101.7	131.5 ± 84.6	0.64 *
Lymphocyte count, mean ± SD, mcL	1451.9 ± 3320.1	1198.6 ± 1095.7	0.40 *	1595.9 ± 4290.1	1228,7 ± 1204.0	0.46 *
**Characteristics at Study Inclusion**						
Delay between first symptoms and inclusion, mean ± SD, days	8.4 ± 4.7	8.3 ± 4.2	0.82 *	8.4 ± 4,8	8.6 ± 4.2	0.84 *
Delay between admission and inclusion, mean ± SD, days	3.0 ± 8.0	3.5 ± 14.6	0.28 ^§^	3.2 ± 8.5	2,2 ± 3.3	0.61 ^§^
SpO_2_/FiO_2_ ratio, mean ± SD,	212.9 ± 41.7	199.4 ± 49.8	0.02 *	210.9 ± 41.9	206.5 ± 48.7	0.53 *
SpO_2_, mean ± SD, %	94.5 ± 3.6	94.0 ± 3.7	0.32 *	94.3 ± 4.0	94.2 ± 3.6	0.84 *
O_2_ flow support, mean ± SD, L/min	8.4 ± 3.4	9.9 ± 5.3	**0.03 ^§^**	8.6 ± 3.4	9,3 ± 4.8	0.59 ^§^
PaO_2_, mean ± SD, mmHg	74.3 ± 25.4	76.8 ± 24.5	0.51 *	75.7 ± 29.0	78.3 ± 24.8	0.59 *
PaCO_2_, mean ± SD, mmHg	36.0 ± 9.1	35.9 ± 7.7	0.93 *	36.3 ± 9.0	35.6 ± 7.6	0.63 *
Systolic blood pressure, mean ± SD, mmHg	130.7 ± 20.0	131.4 ± 20.9	0.8 *	131.3 ± 21.2	130.8 ± 20.3	0.9 *
Temperature, mean ± SD, °C	37.3 ± 1.2	37.3 ± 1.2	0.61 ^§^	37.3 ± 1.3	37.3 ± 1.1	0.55 ^§^
C-reactive protein, mean ± SD, mg/L	144.9 ± 100.7	168.0 ± 95.0	0.07 *	150.8 ± 105.1	163.7 ± 97.9	0.42 *
Lymphocyte count, mean ± SD, mcL	1224.0 ± 2294.0	1128.4 ± 1010.8	0.66 ^§^	1278.8 ± 2907.4	1168.8 ± 1108.5	0.75 ^§^
eGFR, mean ± SD, mL/min/1.73 m^2^	72.6 ± 34.7	152.2 ± 701.9	0.25 *	75.2 ± 34.5	171.8 ± 788.2	0.27 *
Use of non-invasive ventilation or high flow oxygenotherapy after inclusion	5 (3.6%)	8 (7.5%)	0.17	4 (4.8%)	4 (4.8%)	1.0

Data are presented as a number (percentage), unless otherwise noted. Comparison methods were noted: * for Student; ^†^ for Chi-2; ^‡^ for Fischer and ^§^ for Mann–Whitney test. Significant intergroup differences are in bold. Abbreviations: SD: Standard deviation; O_2_: oxygen; ACEI: angiotensin conversion enzyme inhibitor; ARB: angiotensin II receptor blocker; BMI: body-mass index; eGFR: estimated glomerular filtration rate by modification of diet in renal disease (MDRD) formula; FiO_2_: fraction of inspired oxygen; HIV: human immunodeficiency virus; IQR: interquartile range; SpO_2_: pulse oximetry O_2_ saturation. Inclusion is defined as when patients present severity criteria for COVID-19 pneumonia, as defined in the Methods section.

## Supplementary Materials

Supplementary materials are available online at https://www.mdpi.com/1424-8247/13/10/317/s1.
