# Supplementary material for: Effect of Tocilizumab in Hospitalized Patients with Severe COVID-19 Pneumonia: A Case-Control Cohort Study
Source: Pharmaceuticals (Basel). 2020 Oct 17;13(10):317. doi: 10.3390/ph13100317 (PMC7603074; doi:10.3390/ph13100317)
Supplement: Supplementary file 1 [file pharmaceuticals-13-00317-s001.pdf]

Supplementary Material

For "Effect of tocilizumab in hospitalized patients with severe pneumonia COVID-19: a cohort study" by Benjamin Rossi* & Lee S Nguyen* et al.

Supplementary figure. Flow chart

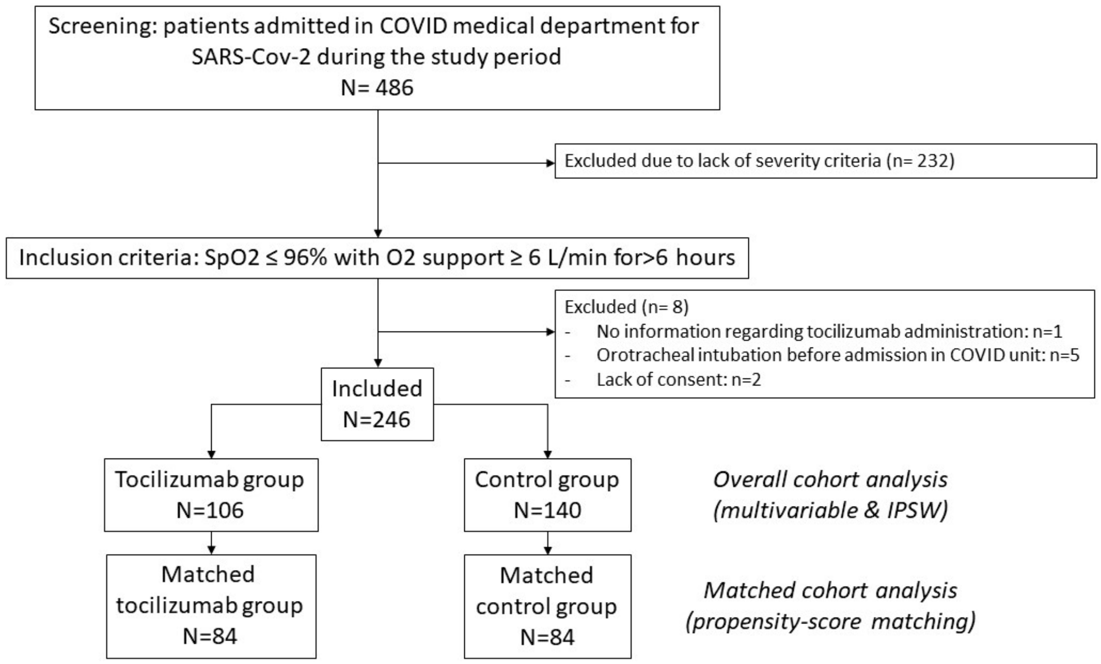

Supplementary Table. Baseline characteristics in the overall cohort and corresponding available data

**Clinical features, No (%)**		
Tocilizumab	106 (43.1%)	all
Age, mean±SD, years	68.0 ±15.0	all
Female	95 (38.6%)	all
Full engagement status	145 (58.9%)	all
Diabetes	101 (41.1%)	all
Insulin treatment	40 (16.3%)	all
Hypertension	143 (58.1%)	all
ACEI treatment	37 (15.0%)	all
ARB treatment	50 (20.3%)	all
Smoker (active or past)	62 (25.2%)	all
History of cardiovascular disease, stroke, peripheral artery disease, heart failure	68 (27.6%)	all
Stroke	26 (10.6%)	all
Peripheral artery disease	8 (3.3%)	all
Heart faillure	11 (4.5%)	all
Atrial fibrillation	30 (12.2%)	all
History_COPD_asthma_emphysema_fibrosis	39 (15.9%)	all
COPD	18 (7.3%)	all
Asthma	15 (6.1%)	all
Emphysema	11 (4.5%)	all
Fibrosis	3 (1.2%)	all
Chronic kidney disease (eGFR<60 ml/min/1,73m^2)	41 (16.7%)	all
Solid organ transplantation	3 (1.2%)	all
Cirrhosis	0 (0.0%)	all
HIV	2 (0.8%)	all
Immunosuppressant drugs	17 (6.9%)	all
Long-term oral corticosteroids	18 (7.3%)	all
Malignancy (active)	23 (9.3%)	all
Obesity (BMI>30kg/m^2)	74 (30.1%)	all
**Treatments after admission**		
No.(%) under antibiotics	240 (97.6%)	all
Betalactamin	234 (95.1%)	all
Macrolide	191 (77.6%)	all
Others	28 (11.4%)	all
No.(%) under antiviral therapy	194 (78.9%)	all
Hydroxychloroquine	188 (76.4%)	all
Lopinavir/ritonavir	9 (3.7%)	all
Immunosuppressants and/or corticosteroids after admission	90 (36.6%)	all
Baricitinib	19 (7.7%)	all

**Characteristics at admission**		
SpO_2, mean ±SD, %	94.0 ±4.0	all
SpO_2/FiO_2 ratio, mean ±SD,	332.1 ±100.5	all
Oxygen flow, mean ±SD, L/min	4.0 ±4.0	all
PaO_2, mean ±SD, mmHg	70.0 ±22.0	209/246 (85.0%)
Temperature, mean ±SD, °C	37.3 ±1.1	all
C-reactive protein, mean ±SD, mg/L	133.0 ±94.0	all
Lymphocyte count, mean ±SD, mcL	1339.5 ±2543.4	all
Delay between first symptoms and admission, means±SD, days	5.0 ±12.0	all
**Characteristics at inclusion**		
Delay between first symptoms and inclusion, mean ±SD, days	8.0 ±4.0	all
Delay between admission and inclusion, mean ±SD, days	3.0 ±11.0	all
SpO_2, mean ±SD, %	94.0 ±4.0	all
O2 flow support, mean ±SD, l/min	9.0 ±4.0	all
Systolic blood pressure, mean ±SD, mmHg	131.0 ±20.0	all
C-reactive protein, mean ±SD, mg/L	154.7 ±98.4	all
Temperature, mean ±SD, °C	37.3 ±1.2	all
Lymphocyte count, mean ±SD, mcL	1182.8 ±1850.9	all
eGFR, mean ±SD, ml/min/1,73m^2	106.9 ±461.9	all
SpO_2/FiO_2 ratio, mean ±SD,	207.1 ±45.7	all
Lymphocyte count, mean ±SD, mcL	1339.5 ±2580.5	239/246 (97.2%)
SpO_2, mean ±SD, %	95.0 ±3.0	163/246 (66.3%)
Delay between inclusion and tocilizumab	1.0 ±1.0	106/106 (all)
PaO_2, mean ±SD, mmHg	75.0 ±25.0	186/246 (75.6%)
C-reactive protein, mean ±SD, mg/L	155.0 ±99.0	all
Use of non-invasive ventilation or high flow oxygenotherapy after inclusion	13 (5.3%)	all

Data are presented as number (percentage), unless otherwise noted.
Abbreviations: O_2: oxygen; ACEI: angiotensin conversion enzyme inhibitor; ARB: angiotensin II receptor blocker; BMI: body-mass index; eGFR: estimated glomerular filtration rate by modification of diet in renal disease (MDRD) formula; FiO_2: fraction of inspired oxygen; HIV: human immunodeficiency virus; IQR: interquartile range; SpO_2: pulse oximetry O_2 saturation

Sensitivity analyses

Cox multivariable models in the dataset of 204 patients (which excluded patients who presented the outcome during the first 48 hours after inclusion).

**Primary outcome**	Adj.HR	95CI	p-value
Tocilizumab	0.402	0.40-0.23	0.001
Full engagement status	0.985	0.98-0.98	<0.001
SpO_2/FiO_2 ratio (per 1-unit increase)	0.398	0.40-0.23	0.001

**Death outcome**	Adj.HR	95CI	p-value
Tocilizumab	0.356	0.18-0.70	0.003
Full engagement status	0.030	0.01-0.12	<0.001
SpO_2/FiO_2 ratio (per 1-unit increase)	0.985	0.98-0.99	<0.001
